# Photoactive Herbal Compounds: A Green Approach to Photodynamic Therapy

**DOI:** 10.3390/molecules27165084

**Published:** 2022-08-10

**Authors:** Cheruthazhakkat Sulaiman, Blassan P. George, Indira Balachandran, Heidi Abrahamse

**Affiliations:** 1 Phytochemistry Division, Centre for Medicinal Plants Research, Arya Vaidya Sala, Kottakkal 676503, India; 2 Laser Research Centre, Faculty of Health Sciences, University of Johannesburg, Doornfontein 2028, South Africa

**Keywords:** photodynamic therapy, plants, phytochemicals, photosensitizers, herbal medicine, natural products

## Abstract

Photodynamic therapy (PDT) is a minimally invasive, alternative, and promising treatment for various diseases, including cancer, actinic keratosis, Bowen’s disease, macular degeneration, and atherosclerotic plaques. PDT involves three different components, photosensitizers (PS), molecular oxygen, and light. The photoactivation of administered PSs using a specific wavelength of light in the presence of molecular oxygen leads to the generation of reactive oxygen species that leads to tumour cell death. Photosensitizing potentials of many commercially available compounds have been reported earlier. However, the possibilities of PDT using herbal medicines, which contain many photosensitizing phytochemicals, are not much explored. Medicinal plants with complex phytochemical compound mixtures have the benefit over single compounds or molecules in the treatment of many diseases with the benefit of low or reduced toxic side effects. This review emphasizes the role of various herbal medicines either alone or in combination to enhance the therapeutic outcome of photodynamic therapy.

## 1. Introduction

Complex phytochemical compounds and mixtures from plants have an advantage over single compounds or molecules for the treatment of many diseases, with reduced or minimal side effects. The World Health Organization (WHO) has, thus, taken cognizance of this new perspective and has recently come out with new guidelines on the technical and procedural standards of alternative and complementary medicine. The therapeutic activity of herbal formulations depends on their phytochemical constituents. Herbal medicines or extracts from plants contain various chemical constituents that could act singly or synergistically. Scientific studies have revealed that the chemical profile of a finished formulation could be different from that of its theoretically anticipated profile. The phenomenon of positive herb–herb interaction, known as synergism, plays an important role in the finished formulations. A study conducted in a classical polyherbal formulation prepared out of *Terminalia chebula*, *Tinospora cordifolia*, and *Zingiber officinale* showed synergetic interaction and chemical transformations of many compounds. Various chromatographic and mass spectroscopic studies revealed that the majority of the compounds present in single extracts were not present in the formulation. Most of the compounds in the formulation were found to be extracted from *Terminalia chebula* and *Tinospora cordifolia*, while *Z. officinale* imparted the enhanced extraction of many compounds from the other two ingredients [1]. The increasing acceptance of Ayurveda, a traditional Indian medicine, across the globe is mostly due to its holistic approach to the therapies. Several countries, including the Middle East, Europe, and the United States, have recognized the Ayurvedic medicinal practice, and many other nations are on the edge of implementing this health care system. Reports say that nearly two-thirds of individuals from the United States use one or more alternative or complimentary treatment modalities, mostly herbal products or plant-based drugs [2,3].

**Figure 1:**
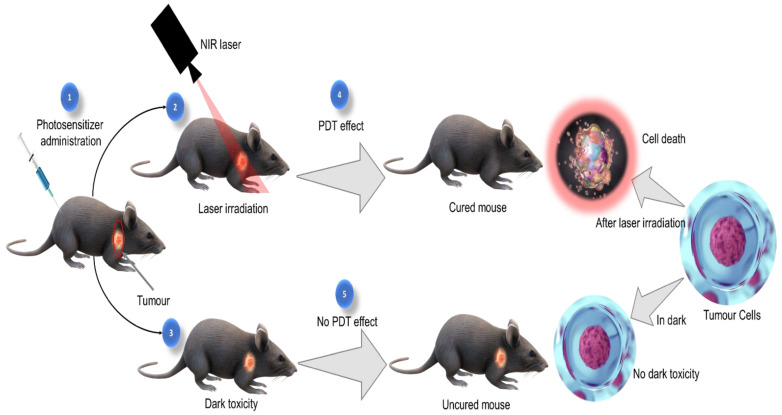
Photodynamic therapeutic effect.

Photodynamic therapy (PDT) is a novel, clinically approved, and minimally invasive treatment modality, used for managing many diseases, including various types of cancers. PDT is based on the application of a non-reactive drug called photosensitizers (PSs), which is accumulated in tumour tissues upon administration. The PS molecules absorb the light of specific wavelength and becomes activated which eventually leads to the generation of reactive oxygen species (ROS) to induce selective destruction of cancer cells [4,5] (Figure 1). In recent years, natural photosensitizers are being used for PDT either alone or in combination with other photosensitizers. Many photoactive compounds were reported from various medicinal plant species that are equally effective as conventional photosensitizers. These studies recommend that herbal medicines with photosensitizing phytochemicals can be used as alternatives to conventional PSs used in PDT [6,7].

Herbal plants and plant extracts are natural compounds and considered as green compared to synthetic chemicals. Due to the environmental sustainability and reduced side effects, in recent years there has been a movement towards the use of natural substances and herbal drugs instead of synthetic chemotherapeutic drugs. Plants with phototoxic compounds were discovered in various families, however the great challenge is their safety, regulatory approval, and demonstration of equal effectiveness compared to synthetic remedies [8]. Phototherapy has reached new heights with the technological advances of the past decades, such as using lasers as the light sources, which are more powerful and controllable. This can be a component of combination therapy for enhanced cancer therapy outcomes. Plants, as a source of PSs, began to be studied three decades ago. Lee et al. reported a new PS consisting of chlorophyll derivates (CpD) from plants. Afterward, the photosensitizing efficacy of CpD was compared with that of a hematoporphyrin derivative (HpD), showing that CpD was as effective as HpD [9]. In this review, the possibilities of PDT using herbal medicines, which contain many photosensitizing phytochemicals, have been discussed.

## 2. Photosensitizers

Photodynamic therapy (PDT) or photochemotherapy is a novel cancer treatment strategy. PDT outcome is based on the administration of a non-toxic drug called photosensitizer (PS). To date, many photosensitizers are approved for clinical use. PS contains various structural groups, including porphyrins and/or their precursors. The PS of therapeutic use mostly gets excited with a specific wavelength preferably between 600 and 800 nm and termed the therapeutic or optical window. An ideal PS exhibits several optimal photochemical and photophysical properties, such as the high absorption peak/wavelength between 600 and 800 nm (red region), should be a single pure compound with good stability and low manufacturing cost, tumour selectivity, minimal systemic toxicity, high potency, etc. The efficacy of an ideal PS depends on the quantum yield of triplet state formation and lifetime which helps in the production of substantial ROS to exert the cellular effect leading to cell death [4].

The commonly used PS of anticancer therapeutic application has a tetrapyrrole-based structure. The PSs with absorption peak in red to deep-red region absorption, such as chlorins, bacteriochlorins, and phthalocyanines, offer better tumour control probability due to deeper penetration into tumour tissue. The minimal dark toxicity and rapid tumour clearance thereby reducing the phototoxic side effects are other important features of an ideal PS. The time duration between the administration of PS and specific wavelength laser irradiation are mostly long to give the PS accumulation in tumour cells [10]. Some reports also support that distinct inflammatory responses and necrosis after laser irradiation is an important immune-stimulating function of PDT, whereas some reports propose that PSs that induce more apoptotic cell death and less inflammatory responses are appropriate for some anticancer applications [11]. Certain PDT mediated apoptosis is immunogenic, and they are able to drive antitumour immunity. The light-induced demolition of the PS is termed as photobleaching, initially thought to be detrimental, but other researchers suggest that this will make light dosimetry less dangerous, as overtreatment is avoided when the PS is demolished through the laser irradiation [12].

Photosensitizers upon laser irradiation capture the light energy and convert it into chemical energy in the presence of molecular oxygen to generate singlet oxygen (^1^O_2_) or superoxide (O_2_) for inducing cellular damage via direct and indirect toxicity [13]. Therefore, the PS is an important element in all PDT events. Several PSs were discovered in the 1980s and 1990s. PS can be classified and divided into three distinct groups based on their chemical structures: (i) porphyrin-based PS (e.g., Photofrin), (ii) chlorophyll-based PS (e.g., chlorine), and (iii) dye (e.g., phthalocyanine). Most of the currently approved PSs in clinical trials belong to the first group. The porphyrins are first generation PSs while porphyrin derivatives are second-generation PSs whereas the third-generation PSs are the modifications of first- and second-generation PSs with biological couples (e.g., antibody conjugate) [14].

## 3. Mechanism of Photodynamic Therapy and Photochemical Reactions

In general, the mechanism of PDT involves the administration of a PS, which selectively accumulates in the target cells, followed by the laser irradiation of the lesion/tumour with the specific wavelength light. The combined effect of two non-reactive and non-toxic components, such as the PS and laser light, with the presence of molecular oxygen leads to the selective killing of target tissue. When PSs are irradiated by the light of the desired wavelength, they absorb light energy and transfer it into molecular oxygen. The energy transferred by the PS converts oxygen in the ground triplet state into singlet state. The singlet-state oxygen is the extremely reactive species which finally leads to the destruction of the target cancer cells [15,16].

**Figure 2:**
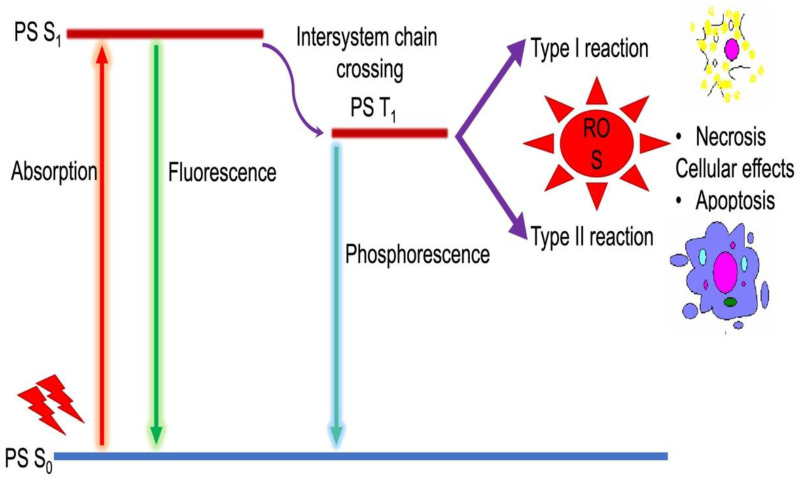
Mechanism of photochemical reactions (Modified Jablonski diagram).

Photodynamic therapy comprises of interaction among three elements: photosensitizer (PS), light, and oxygen. PDT has a unique mechanism of action compared to conventional treatments. The interaction between the photosensitizer, light, and molecular oxygen exerts the PDT cellular effect. A series of Chemico-biological events are involved in the PDT mechanisms. The photochemical mechanism/pathway in PDT is quite complex and a series of energy transfer events that happens during this event is well explained by the Jablonski diagram (Figure 2). After the PS is exposed to the specific wavelength light, the outermost electron in the molecular orbital is excited from the ground (S^0^) to the short-lived excited state (S^1^). The intersystem crossing then transits the molecule to an excited triplet state (T^1^) which has a longer lifetime [17,18]. The PS, in both S^1^ and T^1^ states are very unstable and loses its energy by emission of fluorescence or phosphorescence, and by internal conversion to heat. A PS in the T^1^ state may react photochemically in either Type I or II reaction. In Type I reaction, the excited PSs react with molecular oxygen by electron transfer and leads to the generation of free radicals [13]. The free radicals rapidly interact with biomolecules and result in their destruction. Whereas the Type II reactions occur by direct energy transfer from PS at T^1^ state to the ground state oxygen molecule, results in the generation of ground state S^0^ PS, and excited-state singlet oxygen (^1^O_2_) which is a powerful oxidizing agent [15,19].

## 4. Natural Product Derived Photosensitizers

Natural products are essential sources for drug discovery. Since ancient times, people have relied on herbal remedies for various health care needs. Around 55% of the drugs available in the market are derived from natural products, and over 60% of the currently available anti-tumour agents are of natural products origin [20,21]. Various phytocompounds have been used as chemotherapeutic drugs and photosensitizing agents. Haemoglobin is the first approved phototherapeutic drug, and most of the subsequent photosensitive agents are derivatives of the haemoglobin structure. Natural products/compounds exhibit various structural diversities and biological properties hence used for the discovery of new photosensitizers. Natural product-mediated PDT has a long history, in ancient Egypt, India, Greek, and China people used sunlight exposure for skin diseases [22]. Some of the important features of an ideal PS include that it should be soluble and stable with absorption coefficient between 600 and 800 nm wavelength light, low dark toxicity, selectivity in target tissues, short drug light interval, non-toxicity on healthy tissues, and should be a pure easily available compound [15].

Medicinal plants have been reported to contain PS components with anti-tumour potential. Plants such as *Heterophyllaea pustulata*, *Scutellaria barbata*, *Chelidonium majus*, *Echinops latifolius*, *Aglaonema simplex*, *Annona purpurea*, and *Helleborus niger* are well known for their chemical constituents with photosensitizing activity. Natural compounds such as Hypocrellin A, Phthalocyanine, Tolyporphin, Hypericin, Carvacrol, Pheophorbide A, etc., are reported to possess photodynamic activity with various cellular targets [23,24].

Porphyrins are one of the oldest photosensitizers of natural origin. The hematoporphyrin is the first reported porphyrin compound, a major haemoglobin constituent. In 1911, the photodynamic effect of hematoporphyrin was revealed, and in 1913 its photosensitizing nature in a human was reported [25]. Protoporphyrin IX (PpIX) is another nature-derived porphyrin-based photosensitizer with low systematic toxicity, which is a vital precursor to biologically important prosthetic groups (e.g., heme and cytochrome c) [26,27]. Hematoporphyrin monomethyl ether (HMME, Haemoporfin) is a novel porphyrin-based photosensitizer prepared from haem. Haemoporfin has higher photodynamic activity with low dark toxicity, shorter-term skin photosensitivity, and rapid accumulation in endothelial cells [28].

Chlorins are an important class of photosensitizers of natural products that originated from chlorophyll a. The absorption peak ranges from 650 to 700 nm which helps in tissue penetration. Chlorins PS can also be observed as porphyrin derivatives with two extra hydrogen atoms combined to a peripheral pyrrole double bond. The naturally occurring chlorophyll has a good singlet oxygen generation and strong absorption whereas it lacks water solubility and is unstable, therefore it cannot be used in pharmaceutical applications [29]. The long aliphatic side chain and the central Mg ion removal from chlorophyll a gives Pheophorbide a (PPB*a*), which improves the stability and singlet oxygen generation [30]. The photocytotoxic action of PPB*a* is higher than HpD, and it exhibited higher selectivity and better therapeutic efficacy [31,32]. Talaporfin and Photochlor are derivatives of chlorin PS with improved spectral and chemical properties, such as water-solubility and strong light absorption, high singlet oxygen generation, etc. They are used in the treatment of lung cancer head and neck cancer, skin cancer, oesophageal cancer, and basal cell carcinomas. Photochlor is highly lipophilic [33,34]. Chlorin-based photosensitizers have comparatively longer wavelength absorption, improved tumour selectivity, and lower skin photosensitivity. These enhanced properties encourage researchers to screen the plant extracts to identify potential novel plant-derived photosensitizers.

Riboflavin (vitamin B2) is an United States Food and Drug Administration (FDA)-approved safe and nontoxic substance that exists in various leafy vegetables, mushrooms, eggs, cheese, milk, etc. It is considered a promising natural photosensitizer due to the strong wavelength absorption in the ultraviolet radiation-UVA (360 nm) and visible (440 nm) region with high intersystem crossing quantum yield [35]. Many plants belonging to the family of Umbelliferae contain a class of linear and angular furanocoumarin compounds known as Psoralens, which is characterized by a furan ring attached to a coumarin unit. In the past, people employed Psoralens for the treatment of skin diseases upon exposure to sunlight [36]. Phycobillins are natural pigments of linear tetrapyrrole structure originating from marine algae with strong wavelength absorption in the visible region [37]. Phycoerythrin is a fluorescent probe or food dye that could be sensitized to generate singlet oxygen after red light irradiation and employed as a potent photosensitizer [38,39].

Hypericin is isolated from *Hypericum perforatum*, a second generation PS. It has two wide absorption peaks of UV (300–400 nm) and white light (500–600 nm). This may be a disadvantage of Hypericin PS failing in deep tumour treatments as various other second generation PSs have absorption peaks above 600 nm to permit improved tissue infiltration. Nevertheless, based on the UV-A penetration depth it can be employed in the sub-epidermal and dermal skin vasculature and has potential in clinical practice for the treatment of squamous and basal cell carcinomas, pancreatic, bladder, nasopharyngeal, and skin cancers [40,41,42,43,44].

Natural hypocrellins, primarily hypocrellin A (HA) and hypocrellin B (HB), are another important plant-derived PS isolated from *Hypocrella bambusae*. HA can be converted to HB under specific conditions. Both HA and HB were used in folklore medicine for treating psoriasis, vitiligo, and other related skin disorders. Hypocrellins exhibit great physiochemical properties, such as high singlet oxygen generation, strong photodynamic activity, low dark toxicity, etc., and make them an interesting lead compound in the development of novel plant-derived photosensitizers [45,46].

## 5. Medicinal Plants with Photosensitizing Compounds

**Figure 3:**
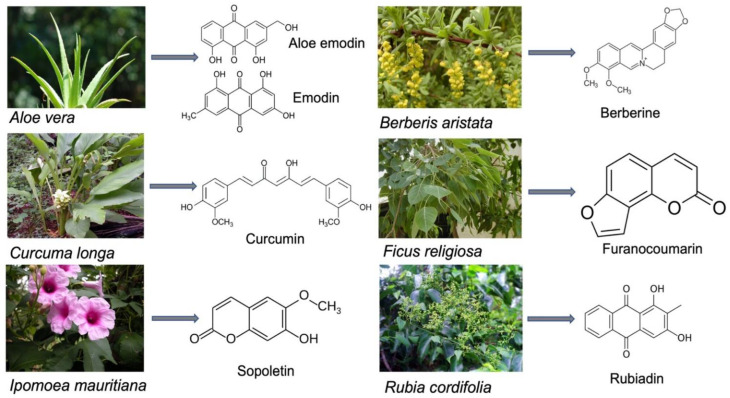
Photosensitizing compounds from medicinal plants.

Traditional medicinal systems have been in practice in India since ancient times. About 70% of the rural populations of India rely on traditional healthcare medicine. In the Indian traditional system of medicine, many plants are used, and it was estimated that more than 5000 medicinal plants are being used by various Indian traditional systems of medicine, such as Ayurveda, Siddha, Unani, and Amchi [45]. In India, about 70% of modern drugs are discovered from natural resources, including the natural product analogs based on bioactive compounds isolated from medicinal plants. It was reported that the majority of cancer drugs available in the market or under research are based on natural products. Currently, about 80% of the antimicrobial, immunosuppressive, cardiovascular, and anticancer drugs are derived from plant-derived sources. More than 70% of entities among 177 anticancer drugs approved are based on natural products or mimetics. It was also estimated that 11% of the total 252 drugs found in the essential medicine list of WHO exclusively originated from plants [46]. Figure 3 shows a list potent medicinal plants and the bioactive compounds from them which possess photosensitizing properties.

### 5.1. Aloe vera (L.) Burm.f.

*Aloe vera* is an important gel-bearing medicinal plant belonging to the family Xanthorrhoeaceae. Though it is native to Africa *A. vera* is also now broadly distributed in many other continents, such as South America, Europe, West Indies, and Asia. Leaf gel contains many phytoconstituents, including vitamins, minerals, amino acids, enzymes, mono and polysaccharides, anthraquinones, phenolics, saponins, lignin, and salicylic acid, which are reported to possess numerous biological properties. Anthraquinones and tricyclic aromatic quinines are the major secondary metabolites of *A. vera* [47,48,49]. Anthraquinones (AQs) are reported to act as photosensitizers capable of producing electronically excited molecules (^1^O_2_) through a Type II reaction or the electronic ground state of the radical anion (O_2_^•−^) through a Type I reaction. In the electronically excited stage, they tend to deactivate by releasing excess energy in the form of heat [50]. AQs like emodin and aloe-emodin are reported to possess PDT action through Type-I and Type-II reactions [7].

### 5.2. Berberis aristata DC.

*Berberis aristata* (Berberidaceae) is an important medicinal plant native to India and Nepal. It is distributed throughout the Himalayas, and also in Sri Lanka. It is extensively used in Ayurveda for treating diarrhoea, jaundice, skin diseases, syphilis, chronic rheumatism and urinary bladder disorders [51,52]. Berberine, an isoquinoline alkaloid, is the major chemical constituent of *B. aristata*. The photosensitizing property of berberine for PDT, in particular, due to their behaviour towards LDL as plasma vehicles, has been reported previously [53]. The antineoplastic effect of Berberine-mediated PDT on HeLa cells was reported by Liu et al. [54]. PDT with berberine is also found to be effective in cervical carcinoma. The study by Oliveira et al. [55] reported the use of berberine in PDT applications. The berberine-mediated PDT induced the generation of ROS, activation of caspase 3 apoptotic proteins, and leads to the caspase-dependent apoptotic cell death mechanism.

### 5.3. Curcuma longa L.

*Curcuma longa* (Zingiberaceae) was used in ancient times in the Indian traditional system as the cure of many diseases, such as inflammation; infections; and gastric, hepatic, and blood disorders. Curcumin is a major bioactive compound from *C. longa*. Curcumin has been reported to possess many pharmacological activities, such as antioxidant, anti-inflammatory, antimicrobial, antitumour, and hepatoprotective activities [56,57]. Curcumin has also been used extensively in food, drugs, and cosmetics. The photoactive potential of curcumin and curcuminoids has been of great scientific interest for decades [58].

Studies reported that curcumin has phototoxic effects and meets the qualities of an ideal PS. An up to 12 g/kg per day dose of curcumin is found to be biologically safe. The selectivity of the curcumin to the target cells makes the application of it in anticancer research. Numerous tumour cell lines showed a preferential or selective absorption of curcumin than the normal healthy cells. A broad absorption spectrum between 300 and 500 nm and high extinction coefficient makes curcumin a potential PS to induce strong phototoxic effects, even at micromolar doses [59,60].

### 5.4. Ficus religiosa L.

*Ficus religiosa* (Moraceae) is a native of the sub-Himalayan region, Bengal, and central India. However, it has been widely spread across the world through cultivation. It is broadly used in traditional herbal medicine for many ailments related to the central nervous and endocrine system, gastrointestinal tract, and the reproductive and respiratory system [61]. Major chemical constituents of *F. religiosa* are phytosterols, amino acids, furanocoumarins, polyphenolics, tannins, and flavonoids. Furanocoumarins, such as 4-methoxy-7H-furo [3,2-g]chromen-7-one and 4-hydroxy-7H-furo [3,2-g]chromen-7-one, have been reported from *F. religiosa*. PDT with furanocoumarins is reported to be very effective for treating some skin diseases, lymphomas, and autoimmune disorders [62,63,64,65].

### 5.5. Ipomoea mauritiana Jacq.

*Ipomoea mauritiana* (Convolvulaceae) is a medicinal plant used in Ayurvedic and Folkloric medicine. The tuberous root of *I. mauritiana* is sweet, cooling in action, appetizer, galactagogue, rejuvenating, stimulant, carminative, and tonic. It consists of various phytochemicals, including taraxerol, taraxerol acetate, β-sitosterol, scopoletin, 7-O-β-D-glycopyranosyl scopoletin caffeoyl glucose, and 5-methoxy-6,7-furanocoumarin [66,67]. Photodynamic therapy using furanocoumarins is reported to be effective for skin diseases and autoimmune disorders [68]. Coumarins and their derivatives are reported to be powerful PDT agents for both superficial diseases and solid tumours [69].

### 5.6. Rubia cordifolia L.

*Rubia cordifolia* (Rubiaceae) is an important medicinal plant used in Ayurveda. R. cordifolia is reported to be used as a blood purifier, and diuretic and possesses vasodilating properties. It also has various other pharmacological properties, such as antioxidant, calcium channel blocker, antiplatelet, antidiabetic, and antistress. It is also used for leucoderma, ulcers, urinary discharges, jaundice, and piles [70,71]. The important chemical ingredients, such as rubiadin, quinine, iridoids, glycosides, triterpenes, and anthraquinones have been reported from *R. cordifolia* [72,73]. Anthraquinones, including rubiadin from *R. cordifolia*, has been reported to exhibit photosensitizing properties. Photoactivity of rubiadin in treating monolayers and multicellular tumour spheroids has been reported by Cogno et al. [53].

## 6. Phytochemicals as Natural Photosensitizers

Light is an effective tool that has been used in the treatment of various skin diseases since the ancient past. It is known that psoralens from *Psoralea corylifolia* were used in India during the Vedic times. Medicinal plants used in traditional systems of medicine, such as Ayurveda, contain many PS reagents and some of the important phytochemicals which can be used for photodynamic therapy are listed in Table 1. The cost effectiveness, bioavailability, and reduced toxicity of natural compounds promotes the use of such compounds in advanced therapeutic options including PDT. Many of the phytochemicals absorb higher energy with shorter waves in UV region and this blue light or UV can excite the molecule into higher energetic states as red light can [74]. Most commonly used PS have multiple absorption peaks and Soret band in the blue region. Chinolin-alkaloids having an absorption maximum of 331 and 360 nm produce singlet oxygen under UV-irradiation. UV light is the key player behind the Furanocoumarins causing phyto-photo-dermatitis in humans. Antitumor activity of Curcumin based on caspase activation was shown against HaCat cells at a sub-apoptotic dose of UVB irradiation. Several reports confirmed the photo-cytotoxicity of Berberine and related NPs under UV, as well as blue light irradiation. Phototoxic effect of Aloe emodin, the major phytochemical from *Aloe vera*, upon UV-irradiation, on skin fibroblasts is mediated by oxidative damage of nucleic acids [75,76,77].

Specific classes of phytochemicals, such as furanocoumarins, polyacetylenes, thiophenes, benzylisoquinolines, beta-carbolines, anthraquinones, phenalenones, phthalocyanines, etc., having appropriate chromophores which can be better utilized in photopharmaceutics. Exploring natural PS, their chemical properties, natural origin, and photopharmaceutical attributes may lead to the identification of potent PDT choice from nature origin.

## 7. Conclusions and Scope for Future Research

Although many synthetic drugs have been studied for their PDT effect, there has been relatively little focus on medicinal plant extracts or bioactive compounds derived from the plants. Medicinal plant extracts are considered to be safer when compared to synthetic chemicals. In conclusion, this review focuses on the medicinal plants with photosensitizing phytochemicals which can be utilized for PDT in the treatment of various diseases, such as cancer. PDT using plant-based photoactive compounds can be considered a green approach in photodynamic therapy. This review will be a lead for future research in this area to establish scientifically the scope of green photodynamic therapy for treating many chronic diseases.

## Figures and Tables

**Table 1:** Phytochemicals from selected medicinal plants with photosensitizing activity.

Phytochemicals	Source Plant	Absorption Wavelength (nm)	Medicinal Properties	References
Aloe emodin	*Aloe vera*	370 to 500	anticancer, antivirus, anti-inflammatory, antibacterial, antiparasitic, neuroprotective, and hepatoprotective	[77,78,79,80]
Emodin	*Aloe vera*	370 to 500	anticancer, anti-inflammatory, antioxidant, antibacterial, antivirus, anti-diabetes	[78,81,82]
Berberine	*Berberis aristata*	250 to 350	immunomodulatory, antioxidative, cardioprotective, hepatoprotective, and renoprotective	[83,84,85]
Curcumin	*Curcuma longa*	350 to 450	antioxidant, anti-inflammatory, neuroprotective, anticancer, hepatoprotective, and cardioprotective	[86,87,88,89,90]
Furanocoumarin	*Ficus religiosa*	320 to 380	antioxidants, antibacterial, analgesic, anticonvulsive, anticoagulant, hypotensive, antidepressants, antifungal, antiviral, anti-inflammatory, antiallergic	[91,92,93]
Scopoletin	*Ipomoea mauritiana*	250 to 350	antibacterial, antifungal, anti-inflammatory	[94,95]
Rubiadin	*Rubia cordifolia*	350 to 450	anticancer, antiosteoporotic, hepatoprotective, antiepileptic, neuroprotective, anti-inflammatory, antidiabetic, antioxidant, antibacterial, antimalarial, antifungal, and antiviral	[96,97,98]

## Data Availability

Not applicable.
